# Clinical Characteristics, Microbiological Profile, and In-Hospital Outcomes of Community-Acquired Pyogenic Bacterial Meningitis in Adults Across Five Hospitals in Jeddah, Saudi Arabia: A Multicenter Retrospective Cohort Study

**DOI:** 10.7759/cureus.115108

**Published:** 2026-08-24

**Authors:** Hasan M Alshami, Fatemah Alzaghabi, Abdullah Al-Garni, Ibrahim Asiri, Tariq M Alshami

**Affiliations:** 1 Public Health Department, King Abdulaziz Hospital, Jeddah, SAU; 2 Directorate of Health Affairs for Public Health Division, Ministry of Health, Jeddah, SAU; 3 Communicable Disease Control Department/ Administration of Public Health, Ministry of Health, Jeddah, SAU; 4 Public Health Authority, Western Region Branch, Jeddah, SAU; 5 Home Care Medicine, King Abdulaziz Hospital, Makkah, SAU

**Keywords:** adult meningitis, bacterial meningitis, cerebrospinal fluid, community-acquired infection, in-hospital mortality, intensive care, polymerase chain reaction, saudi arabia

## Abstract

Background

Contemporary multicenter data describing adult community-acquired pyogenic bacterial meningitis in Saudi Arabia remain limited. This study characterized the demographic, clinical, microbiological, and in-hospital outcome profile of cases identified across five hospitals in Jeddah.

Methods

We conducted a multicenter retrospective cohort study using surveillance and hospital records from January 1, 2020, to December 31, 2025. Adults with confirmed or probable community-acquired pyogenic bacterial meningitis were included. Microbiological confirmation was established by culture or cerebrospinal fluid (CSF) polymerase chain reaction (PCR), with PCR applied to culture-negative specimens. Descriptive analyses used variable-specific denominators. Exploratory group comparisons were performed using the Mann-Whitney U test and Fisher exact test. Because of the limited sample, multivariable analyses were conducted using Firth penalized logistic regression with adjustment for age and admission Glasgow Coma Scale (GCS) score as covariates.

Results

Among 39 candidate records, five pediatric records and five records with a non-community-acquired source were excluded, leaving 29 adults. No eligible adult cases were identified during 2020-2022. The median age was 41 years, and 13 patients (44.8%) were male. All 29 cases (100.0%) were microbiologically confirmed after including cerebrospinal fluid (CSF) polymerase chain reaction (PCR) results, and PCR alone established the etiology in nine culture-negative cases (31.0%). *Streptococcus pneumoniae* was found in 11 cases (37.9%), *Neisseria meningitidis* in 10 (34.5%), gram-negative bacilli in six (20.7%), and other confirmed pyogenic bacteria in two (6.9%). Intensive care unit (ICU) admission occurred in 19 of 28 patients (67.9%), and nine of 29 patients died in the hospital (31.0%). The median length of stay was 15 days. Patients who died were older than survivors, and a GCS score of eight or lower was associated with mortality in unadjusted analysis. In the limited mortality model, age and GCS did not independently reach statistical significance. Older age remained associated with ICU admission in the exploratory adjusted model.

Conclusions

In this cohort, community-acquired pyogenic bacterial meningitis in adults was associated with high critical care requirements and substantial in-hospital mortality. The combined use of CSF culture and PCR identified an etiology in all patients and provided microbiological confirmation for nine culture-negative episodes. These findings support early assessment of clinical severity, routine use of molecular diagnostics in culture-negative specimens, and surveillance strategies that reflect local etiologic patterns.

## Introduction

Community-acquired bacterial meningitis is a medical emergency associated with progressive neurological deterioration, intensive care requirements, death, and long-term neurological disability [1-3]. Although conjugate vaccination programs and changes in population risk have altered disease epidemiology, clinically important adult disease continues to occur, and the distribution of causative pathogens varies by age, geography, vaccination coverage, and host factors [2,4]. Current clinical guidance emphasizes immediate recognition, proper antimicrobial therapy, microbiological confirmation, and early management of systemic and neurological complications [5].

Saudi national surveillance data provide broad information on reportable bacterial infections but contain limited clinical detail for meningitis [6]. Recent Saudi studies have described meningitis and encephalitis within individual tertiary centers or mixed populations that included pediatric, nosocomial, neurosurgical, or non-bacterial cases [7-9]. These studies are valuable but do not fully characterize adult community-acquired pyogenic meningitis across Jeddah hospitals.

The present study therefore aimed to describe the demographic characteristics, clinical presentation, microbiological profile, notification timeliness, intensive care use, hospital length of stay, and in-hospital mortality of adults with community-acquired pyogenic bacterial meningitis identified across five hospitals in Jeddah, Saudi Arabia. Exploratory analyses also assessed factors associated with in-hospital mortality and intensive care unit admission.

## Materials and methods

Study design and setting

This multicenter retrospective cohort study used routinely collected hospital records and Health Electronic Surveillance Network (HESN) notification records from five hospitals in Jeddah within Jeddah Health Clusters 1 and 2, Saudi Arabia. The prespecified screening period extended from January 1, 2020, through December 31, 2025. Eligible admissions occurred from 2023 through 2025; no eligible adult cases were identified during 2020-2022. The report was prepared in accordance with the Strengthening the Reporting of Observational Studies in Epidemiology (STROBE) statement [10].

Participants and case definitions

We reviewed candidate records. Inclusion required age 18 years or older, inpatient admission during the study period, and a diagnosis of community-acquired pyogenic bacterial meningitis.

Microbiologically confirmed disease required detection of a compatible bacterial pathogen in cerebrospinal fluid (CSF) or blood by culture or polymerase chain reaction (PCR) in a clinically compatible illness. Probable disease required a compatible clinical syndrome and a CSF profile consistent with pyogenic meningitis when microbiological results were negative or unavailable, and no alternative non-bacterial etiology was identified. CSF PCR was performed at the discretion of the treating team, predominantly on culture-negative specimens, and a positive PCR result in a clinically compatible illness was treated as microbiological confirmation on the same footing as culture [2,5].

Records were excluded for age younger than 18 years, fungal or other non-bacterial meningitis, hospital-acquired onset, post-neurosurgical or shunt-related infection, device-related infection, post-traumatic meningitis, duplicate episodes, or insufficient information to establish eligibility. Community-acquired status and the absence of neurosurgical, traumatic, shunt-related, and device-related causes were confirmed by the investigators for the final cohort.

Data sources and variables

Data were abstracted from surveillance notifications and available electronic or paper hospital records. Case finding proceeded from HESN notification records; hospital discharge coding was not independently searched. Laboratory and CSF values were recorded from the measurement closest to the admission date. Variables included age, sex, nationality, selected comorbidities, antibiotic exposure before admission, presenting symptoms, admission Glasgow Coma Scale (GCS) score, peripheral white blood cell (WBC) count, CSF findings, blood and CSF culture results, CSF PCR result and organism detected, causative organism, admission and notification dates, ICU admission, hospital discharge date, and vital status at discharge. Direct identifiers were used only for record linkage and were removed from the analytic dataset.

Outcomes

The primary descriptive outcomes were in-hospital mortality, ICU admission, and hospital length of stay. Secondary outcomes included etiologic distribution, microbiological confirmation, the method of confirmation (culture, PCR, or both), and the number of days from hospital admission to HESN notification, known as notification lag. Neurological complications at discharge were not treated as a principal comparative outcome because documentation was incomplete.

Statistical analysis

Categorical variables were summarized as frequencies and percentages. Continuous variables were summarized as medians and interquartile ranges because of the small cohort and skewed distributions. Exact binomial 95% confidence intervals (CIs) were calculated for the principal proportions. Unknown and not-performed values were excluded variable by variable, and available denominators were reported. No missing values were imputed.

Exploratory comparisons between patients who died and survivors and between patients admitted and not admitted to the ICU used the two-sided Mann-Whitney U test for continuous variables and the two-sided Fisher exact test for categorical variables. Unadjusted odds ratios (ORs) with 95% CIs were reported for binary variables. A 0.5 Haldane-Anscombe correction was applied when a contingency-table cell was zero. Because the cohort and event counts were small, adjusted analyses used Firth penalized logistic regression, which reduces small-sample bias and addresses separation [11,12]. Each model was deliberately restricted to age and admission GCS score. Age was modeled per 10-year increase. All p-values were two-sided, and p < 0.05 was considered statistically significant. Because the comparisons were exploratory and multiple, p-values were not adjusted and were interpreted cautiously. Analyses were performed using Python version 3.11.

Measures to minimize bias

Multiple measures were taken to limit bias. Eligibility criteria and case definitions were prespecified and applied consistently to all candidate records. Community-acquired status was independently verified for each included case, while hospital-acquired, post-neurosurgical, shunt-related, device-related, and post-traumatic episodes were excluded to minimize classification errors of the exposure of interest. Source records were re-examined during data cleaning, and discrepancies in admission, discharge, and notification dates were resolved against the original documents. Derived variables, including length of stay and notification lag, were recalculated from verified dates. Microbiological classification was based on documented laboratory results rather than clinical judgment, and the method of confirmation was recorded separately from the organism to distinguish culture-based from molecular diagnoses. PCR-confirmed cases were further verified against HESN notification records, which incorporate results reported directly by the regional reference laboratory; where the notified record and the hospital chart differed, the organism and confirmation method were updated to match the laboratory-sourced notification record. This provided an independent source for molecular results rather than relying on a single hospital record.

We took several measures to reduce information and analytical bias. Incomplete documented missing values were not imputed. For each variable, we reported denominators separately and excluded unknown or unperformed results from the analysis instead of treating them as negative findings. Given the small sample size, exact binomial confidence intervals were used instead of normal approximations. When a contingency-table cell had zero cases, we applied the Haldane-Anscombe correction. We also used Firth-penalized logistic regression to minimize small-sample bias and address separation. To avoid overfitting, adjusted models included only two prespecified covariates, and all comparative analyses were designated exploratory in advance, with unadjusted p-values interpreted accordingly rather than as confirmatory evidence.

Selection bias arising from notification-based case ascertainment could not be eliminated by design, as hospital discharge coding was not independently reviewed; this residual bias, along with the selective use of CSF-PCR and the recording of laboratory values closest to admission, is addressed in the Limitations.

Ethical considerations

The Local Research Ethics Committee of the Ministry of Health Branch in Jeddah approved the study (approval number A02534; LEC reference number KSA: H-02-J-002; approval date June 4, 2026). The committee approved a waiver of informed consent, as the study involved retrospective review of existing records with a de-identified analytic dataset.

## Results

Cohort assembly

The initial dataset comprised 39 candidate records. Ten records were excluded: five patients were under 18 years of age, two had post-traumatic meningitis, one had a post-neurosurgical infection, one had a shunt-related infection, and one had a device-related infection. Consequently, 29 adults with community-acquired pyogenic bacterial meningitis were included. No eligible adult cases were identified in 2020, 2021, or 2022. In contrast, 5 cases were reported in 2023, 10 in 2024, and 14 in 2025. All 29 cases (100%; 95% CI, 88.1-100.0%) were microbiologically confirmed following the incorporation of CSF PCR results, and no case remained classified as probable pyogenic meningitis.

Demographic and clinical characteristics

The median age of the cohort was 41 years (interquartile range, 32-60 years). Of the patients, 13 (44.8%) were male, and 15 (51.7%) were Saudi nationals. Fever was observed in 23 of 25 patients with available data (92.0%), headache in 16 of 18 (88.9%), neck stiffness in 10 of 13 (76.9%), and altered consciousness in 17 of 25 (68.0%). The median admission GCS score was 12 (interquartile range, 9-14), with seven patients (24.1%) presenting a score of eight or lower. Baseline characteristics are presented in Table 1.

**Table 1:** Demographic, Clinical, Laboratory, and Outcome Characteristics of the Cohort Values are presented as median (interquartile range) or n/N (%). Denominators vary because unknown or not-performed values were excluded variable by variable. GCS, Glasgow Coma Scale; WBC, white blood cell count; CSF, cerebrospinal fluid; PCR, polymerase chain reaction.

Characteristic	Result	Available N
Age, years	41 (32–60)	29
Age 18-29 years	6/29 (20.7%)	29
Age 30-44 years	11/29 (37.9%)	29
Age 45-59 years	4/29 (13.8%)	29
Age 60-74 years	8/29 (27.6%)	29
Male sex	13/29 (44.8%)	29
Saudi nationality	15/29 (51.7%)	29
Diabetes mellitus	8/17 (47.1%)	17
Immunosuppression or human immunodeficiency virus	1/29 (3.4%)	29
Antibiotics before admission	18/29 (62.1%)	29
Fever	23/25 (92.0%)	25
Headache	16/18 (88.9%)	18
Neck stiffness	10/13 (76.9%)	13
Altered consciousness	17/25 (68.0%)	25
Seizure or focal neurological deficit	2/12 (16.7%)	12
Admission GCS score	12 (9–14)	29
Admission GCS score of eight or lower	7/29 (24.1%)	29
Peripheral WBC, x 10^9/L	15.2 (9.0–19.0)	29
CSF glucose, mg/dL	20.0 (7.2–59.8)	29
Blood culture positive	5/26 (19.2%)	26
CSF culture positive	19/28 (67.9%)	28
CSF PCR performed	11/29 (37.9%)	29
CSF PCR positive	11/11 (100.0%)	11
Microbiologically confirmed	29/29 (100.0%)	29
Confirmed by culture alone	18/29 (62.1%)	29
Confirmed by PCR alone	9/29 (31.0%)	29
Confirmed by both culture and PCR	2/29 (6.9%)	29
Intensive care unit admission	19/28 (67.9%)	28
In-hospital mortality	9/29 (31.0%)	29
Hospital length of stay, days	15 (10–47)	29
Notification lag, days	2 (1–3)	29

Microbiological profile

*Streptococcus pneumoniae* was found in 11 cases (37.9%) and *Neisseria meningitidis* in 10 cases (34.5%). Gram-negative bacilli accounted for six cases (20.7%), while two cases (6.9%) involved other confirmed pyogenic bacteria. CSF culture was positive in 19 of 28 patients with available results (67.9%), and blood culture was positive in five of 26 cases (19.2%).

CSF PCR was performed in 11 patients (37.9%) and was positive in all 11. Nine of these patients (31.0% of the cohort) had no organism recovered from CSF or blood culture, so PCR alone established the etiology; the remaining two had concordant culture and PCR results. Every one of the nine culture-negative patients tested by PCR yielded a pathogen, and no episode remained classified as probable pyogenic meningitis. PCR contributed five of the 10 meningococcal cases, three of the 11 pneumococcal cases, and one gram-negative bacillary case. Overall, 18 cases (62.1%) were confirmed by culture alone, nine (31.0%) by PCR alone, and two (6.9%) by both methods. Etiology-specific outcomes are shown descriptively in Table 2.

**Table 2:** Etiologic Distribution and In-Hospital Outcomes The final column shows the number of cases in each group for which PCR alone established the etiology because CSF and blood cultures were negative. No case remained classified as probable pyogenic meningitis after PCR results were incorporated. Etiology-specific comparisons were descriptive because of small cell sizes. ICU, intensive care unit; IQR, interquartile range; PCR, polymerase chain reaction; CSF, cerebrospinal fluid.

Etiology	Cases, n (%)	Deaths, n (%)	ICU admission, n/N (%)	Length of stay, median (IQR), days	Confirmed by PCR alone, n
Streptococcus pneumoniae	11 (37.9%)	3 (27.3%)	6/11 (54.5%)	13 (10-39.5)	3
Neisseria meningitidis	10 (34.5%)	3 (30.0%)	7/10 (70.0%)	14.5 (10-41.2)	5
Gram-negative bacilli	6 (20.7%)	3 (50.0%)	4/5 (80.0%)	34.5 (15.2-50.8)	1
Other confirmed pyogenic bacteria	2 (6.9%)	0 (0.0%)	2/2 (100.0%)	57 (34-80)	0
Total	29 (100.0%)	9 (31.0%)	19/28 (67.9%)	15 (10-47)	9

In-hospital outcomes

ICU status was known for 28 patients, of whom 19 were admitted to the ICU (67.9%; 95% CI, 47.6%-84.1%). Nine patients died in the hospital, yielding a case-fatality proportion of 31.0% (95% CI, 15.3%-50.8%). The median hospital length of stay was 15 days (interquartile range, 10-47 days).

Exploratory mortality analysis

Patients who died were older than survivors (median, 57 versus 37 years; U = 132.5; p = 0.048) and had lower admission GCS scores (median, 8 versus 12; U = 47.5; p = 0.045). A GCS score of eight or lower was associated with higher mortality in unadjusted analysis: five of seven patients (71.4%) with a score of eight or lower died compared with four of 22 (18.2%) with a higher score (OR, 11.25; 95% CI, 1.58-80.30; p = 0.016). Other exploratory comparisons are summarized in Table 3. In the limited Firth model, neither age per 10-year increase (adjusted OR, 1.56; 95% CI, 0.91-2.88; p = 0.106) nor admission GCS per one-point increase (adjusted OR, 0.84; 95% CI, 0.64-1.09; p = 0.176) independently reached statistical significance.

**Table 3:** Exploratory Comparison of Patients Who Died in the Hospital and Survivors Values are median (interquartile range) or n/N (%). Continuous variables were compared with the two-sided Mann-Whitney U test; categorical variables were compared with the two-sided Fisher exact test. Microbiological confirmation could not be compared between groups because all 29 patients were confirmed. All p-values were unadjusted and exploratory. An asterisk (*) denotes a statistically significant p-value (p < 0.05). GCS, Glasgow Coma Scale; WBC, white blood cell count; CSF, cerebrospinal fluid; CI, confidence interval; OR, odds ratio; PCR, polymerase chain reaction.

Variable	Deaths	Survivors	Effect estimate	Test statistic	p-value
Age, years	57 (44-65)	37 (29.2-47.8)	Rank-biserial r = 0.47	U = 132.5	0.048*
Admission GCS score	8 (8-12)	12 (10.8-15)	Rank-biserial r = -0.47	U = 47.5	0.045*
Peripheral WBC, x 10^9/L	11 (4.9-16)	16.4 (12.3-23)	Rank-biserial r = -0.37	U = 56.5	0.12
CSF glucose, mg/dL	44 (20-72)	19 (5-38.8)	Rank-biserial r = 0.37	U = 123.5	0.119
Male sex	6/9 (66.7%)	7/20 (35.0%)	OR 3.71 (95% CI, 0.70-19.59)	Fisher exact	0.226
Saudi nationality	2/9 (22.2%)	13/20 (65.0%)	OR 0.15 (95% CI, 0.02-0.95)	Fisher exact	0.05
Antibiotics before admission	7/9 (77.8%)	11/20 (55.0%)	OR 2.86 (95% CI, 0.47-17.35)	Fisher exact	0.412
Altered consciousness	6/8 (75.0%)	11/17 (64.7%)	OR 1.64 (95% CI, 0.25-10.77)	Fisher exact	1
GCS score of eight or lower	5/9 (55.6%)	2/20 (10.0%)	OR 11.25 (95% CI, 1.58-80.30)	Fisher exact	0.016*
Confirmed by CSF PCR	5/9 (55.6%)	6/20 (30.0%)	OR 2.92 (95% CI, 0.57-14.82)	Fisher exact	0.237
Confirmed by PCR alone (culture negative)	3/9 (33.3%)	6/20 (30.0%)	OR 1.17 (95% CI, 0.22-6.28)	Fisher exact	1

Exploratory intensive care unit analysis

Patients admitted to the ICU were older than those who were not admitted (median, 57 versus 36 years; U = 128.5; p = 0.036). In the limited adjusted model, age remained associated with ICU admission (adjusted OR, 2.04 per 10-year increase; 95% CI, 1.10-4.65; p = 0.021), whereas admission GCS did not (adjusted OR, 1.02 per one-point increase; 95% CI, 0.79-1.32; p = 0.902). Exploratory unadjusted comparisons are shown in Table 4. The observed nationality association was considered vulnerable to confounding by age, hospital, referral pathway, and other unmeasured case-mix factors and was not interpreted as a biological effect.

**Table 4:** Exploratory Comparison by Intensive Care Unit Admission One patient with unknown intensive care unit status was excluded. Values are median (interquartile range) or n/N (%). Microbiological confirmation could not be compared between groups because all 29 patients were confirmed. All p-values were unadjusted and exploratory. The nationality result should not be interpreted as a biological association. An asterisk (*) denotes a statistically significant p-value (p < 0.05). GCS, Glasgow Coma Scale; WBC, white blood cell count; CSF, cerebrospinal fluid; CI, confidence interval; ICU, intensive care unit; OR, odds ratio; PCR, polymerase chain reaction.

Variable	ICU admission	No ICU	Effect estimate	Test statistic	p-value
Age, years	57 (34.5-62)	36 (32-40)	Rank-biserial r = 0.50	U = 128.5	0.036*
Admission GCS score	11 (8-12.5)	12 (10-15)	Rank-biserial r = -0.23	U = 66.0	0.344
Peripheral WBC, x 10^9/L	14 (5.8-20.6)	16.8 (13.2-19)	Rank-biserial r = -0.22	U = 67.0	0.376
CSF glucose, mg/dL	32 (6.1-65.9)	20 (16.2-32)	Rank-biserial r = 0.22	U = 104.0	0.375
Male sex	10/19 (52.6%)	3/9 (33.3%)	OR 2.22 (95% CI, 0.43-11.60)	Fisher exact	0.435
Saudi nationality	6/19 (31.6%)	8/9 (88.9%)	OR 0.06 (95% CI, 0.01-0.57)	Fisher exact	0.013*
Antibiotics before admission	14/19 (73.7%)	4/9 (44.4%)	OR 3.50 (95% CI, 0.66-18.50)	Fisher exact	0.21
Altered consciousness	13/17 (76.5%)	4/8 (50.0%)	OR 3.25 (95% CI, 0.55-19.32)	Fisher exact	0.359
GCS score of eight or lower	6/19 (31.6%)	1/9 (11.1%)	OR 3.69 (95% CI, 0.37-36.57)	Fisher exact	0.371
Confirmed by CSF PCR	7/19 (36.8%)	4/9 (44.4%)	OR 0.73 (95% CI, 0.15-3.65)	Fisher exact	1
Confirmed by PCR alone (culture negative)	5/19 (26.3%)	4/9 (44.4%)	OR 0.45 (95% CI, 0.08-2.36)	Fisher exact	0.407
In-hospital mortality	9/19 (47.4%)	0/9 (0.0%)	OR 17.19 (95% CI, 0.88-337.16)	Fisher exact	0.026*

Surveillance timeliness

All cases in the available analytic dataset had a corresponding HESN notification. The median notification lag was two days (interquartile range, one to three days), and no case was notified more than seven days after admission. Because the available dataset did not include hospital-only non-notified cases, notification completeness could not be estimated.

## Discussion

This multicenter retrospective cohort described 29 adults with community-acquired pyogenic bacterial meningitis identified across five hospitals in Jeddah. All cases were microbiologically confirmed after accounting for CSF PCR, and *S. pneumoniae* and *N. meningitidis* were the leading pathogens, together accounting for just over seven-tenths of the cohort. Use of critical care resources was common, and approximately one-third of the cohort died during hospitalization. Older age and severe impairment of consciousness were associated with adverse outcomes in unadjusted analyses, although the limited mortality model did not identify an independently significant association after simultaneous adjustment for age and GCS. These results should be viewed as descriptive and hypothesis-generating due to the small cohort size and incomplete documentation of several covariates.

The observed case-fatality proportion of 31.0% was higher than the 21% mortality reported in the landmark Dutch prospective cohort and was also substantial compared with contemporary nationwide community-acquired cohorts [3,13]. It is difficult to make direct comparisons because of differences in pathogen distribution, referral patterns, age demographics, treatment approaches, and how outcomes are defined. The wide confidence interval around the current estimate further underscores the imprecision associated with the small sample. Additionally, since many patients were admitted to intensive care, the group studied mostly identified a cohort with severe illness in the hospital, not everyone in the community.

Advanced age and decreased level of consciousness are well-known prognostic markers in adult bacterial meningitis [3,14]. In the present cohort, a GCS score of eight or lower showed a strong unadjusted linkage with mortality, but the confidence interval was wide and not statistically significant for GCS as a continuous variable in the restricted adjusted model. This pattern is compatible with limited power, sparse events, and possible nonlinearity in the relationship between consciousness level and outcome. The exploratory association between age and ICU admission similarly needs to be confirmed in larger studies.

The predominance of pneumococcal disease, with meningococcal disease close behind, is broadly consistent with international adult cohorts in which S. pneumoniae leads, although the meningococcal share in the present cohort was higher than in many of those series [1,2,4]. Gram-negative bacilli still accounted for six cases (20.7%), a notable proportion for community-acquired disease, and this figure was reached only after post-neurosurgical, post-traumatic, shunt-related, and device-related episodes had been excluded, which removed several gram-negative infections. The residual gram-negative burden is therefore unlikely to be explained by unrecognized healthcare-associated cases, although etiologic subgroups were too small for reliable inferential comparisons. Saudi single-center studies have reported heterogeneous pathogen distributions, including significant gram-negative disease, but those cohorts often combined adults and children or included nosocomial and neurosurgical infections [7-9]. Restriction to adult community-acquired pyogenic disease therefore addresses a different clinical population, which may explain some of the variation.

The contribution of molecular testing was the most striking microbiological finding. CSF PCR was positive in all 11 patients in whom it was performed and provided the only etiologic diagnosis in nine patients whose cultures were negative, eliminating the probable-case category entirely. Because PCR was requested mainly after cultures returned negative, this yield reflects targeted rather than universal testing and should not be seen as the yield from routine molecular testing of every patient.

The direction of the finding is nonetheless consistent with guideline recommendations and with reports that nucleic acid amplification substantially increases etiologic ascertainment when specimens are collected after antibiotic exposure [2,5]. This is directly relevant here, because 18 of 29 patients (62.1%) had received antibiotics before admission, an exposure that suppresses culture yield while leaving bacterial nucleic acid detectable. Systematic PCR testing of all CSF specimens with a pyogenic profile would likely have identified further pathogens among the patients who were never tested, and the reclassification of five meningococcal and three pneumococcal cases as confirmed disease has direct implications for chemoprophylaxis of contacts and for vaccine-preventable disease surveillance.

Fever, headache, neck stiffness, and altered consciousness were common when documented, consistent with established descriptions of adult bacterial meningitis [1,2]. Incomplete symptom documentation precluded calculation of complete-cohort prevalence for several classic clinical findings.

The frequent use of antibiotics before admission may also have affected culture yield, although CSF culture positivity remained moderate among patients with known results and molecular testing recovered an etiology in every culture-negative patient in whom it was attempted. Prompt antimicrobial therapy remains central to management, and treatment delay has been associated with worse outcomes in community-acquired bacterial meningitis [5,15]. The present dataset did not contain reliable time-to-first-antibiotic information, so this clinically important relationship could not be assessed.

The surveillance component showed that all included cases were notified, with a median delay of two days. This finding describes timeliness only and does not establish completeness, as the dataset lacked a denominator of hospital-identified cases without HESN notification. National surveillance initiatives have similarly emphasized the need for more comprehensive clinical and outcome variables to strengthen interpretation of reportable disease data [6]. Integrating hospital case-finding, microbiology databases, and HESN records would enable a more rigorous assessment of notification completeness and delays.

Limitations

This study had several limitations. First, the cohort was small, drawn from five hospitals, and contained only nine deaths, which limited precision and the number of covariates that could be included in adjusted models; small microbiological subgroups likewise limited pathogen-specific outcome comparisons. Second, missing data were substantial for several symptoms, comorbidities, imaging findings, and neurological complications, and the use of available-case denominators may have introduced information bias. Some prespecified variables were also unavailable, including a validated comorbidity index, time from admission to effective antimicrobial therapy, and detailed discharge disposition. Third, case identification was based solely on HESN notifications. Hospital discharge databases and ICD-10 codes were not reviewed, which could have missed patients who were treated but not included in the sampling frame. The cohort therefore represents notified rather than hospitalized disease; the true case number is likely higher than 29. The same reasoning applies to the absence of eligible cases during 2020-2022, which may reflect notification behavior during the coronavirus disease 2019 response rather than true absence of disease. Fourth, laboratory and CSF values were taken from the measurement closest to admission and therefore capture presentation rather than trajectory; they should not be read as peak severity, which may partly explain why associations between admission laboratory values and outcome were weak. Fifth, 18 of 29 patients (62.1%) received antibiotics before admission. Antibiotics can sterilize CSF within hours. As a result, culture may be falsely negative despite a diagnostic clinical and CSF picture. More prolonged pre-admission treatment may attenuate derangements in CSF cell count, protein, and glucose. This plausibly explains part of the culture-negative fraction that molecular testing resolved and means the reported CSF parameters may understate pre-treatment severity; neither agent nor timing was recorded, so the effect could not be quantified. Sixth, CSF PCR was performed in only 11 of 29 patients and directed mainly at culture-negative specimens. Finally, the retrospective observational design precluded causal inference. Exploratory p-values were unadjusted for multiple comparisons. The cohort included only hospitalized cases from participating sites, so it should not be read as a population-based incidence estimate for Jeddah.

## Conclusions

This multicenter cohort provides a focused description of adult community-acquired pyogenic bacterial meningitis in Jeddah. The disease was associated with substantial critical care use and in-hospital mortality, while older age and impaired consciousness emerged as clinically important signals for severity. Incorporating CSF PCR alongside culture established a microbiological diagnosis in every patient and identified an organism in nine culture-negative episodes, supporting routine molecular testing of culture-negative CSF in this setting. The heterogeneous microbiological profile supports continued local surveillance and timely organism-directed management. Future work should use prospective multicenter case ascertainment, protocolized molecular testing of all eligible CSF specimens, standardized clinical documentation, complete treatment timing, and structured neurological outcome assessment to produce more precise estimates and clinically useful risk models.
